# Prevalence, correlates, and mortality impacts of ventricular arrhythmia among older men and women: a population-based cohort study in Moscow

**DOI:** 10.1186/s12872-021-01883-0

**Published:** 2021-02-08

**Authors:** Maria A. Shkolnikova, Rukijat A. Ildarova, Dmitri A. Jdanov, Svetlana A. Shalnova, Vladimir M. Shkolnikov

**Affiliations:** 1 Centre for Cardiac Arrhythmia, Pirogov Russian National Research Medical University, Taldomskaya 2, Moscow, Russian Federation 125412; 2 Max Planck Institute for Demographic Research, Konrad-Zuse-Str. 1, 18057 Rostock, Germany; 3 National Research University Higher School of Economics, Bol’shoj Trehsvjatitel’skij Pereulok, Moscow, Russian Federation 109028; 4 National Research Center for Preventive Medicine, Petroverigskiy pereulok 10, Moscow, Russian Federation 101990

**Keywords:** Cardiovascular mortality, Male–female mortality difference, Old age, Ventricular arrhythmia, Ventricular premature complexes, Holter monitoring, ECG

## Abstract

**Background:**

In Russia, cardiovascular disease (CVD) mortality is high and the mortality gap between men and women is large. Conventional risk factors cannot explain these phenomena. Ventricular arrhythmia (VA) is an important contributor to the death toll in community-based populations. The study examines the prevalence and the mortality impacts of VA in men and women and the role of VA in the male mortality excess at older ages.

**Methods:**

This is a secondary analysis of data from the Stress, Aging, and Health in Russia (SAHR) study that was fielded in 2007–9 in Moscow (1800 individuals, mean age 68.8 years), with mean mortality follow-up of 7.4 years (416 deaths, 248 CVD deaths). Indicators reflecting the frequency and the complexity of VA were derived from 24-h ambulatory ECG recordings. Other covariates were: socio-demographic characteristics, conventional risk factors, markers of inflammation, reported myocardial infarction, and stroke. The impacts of VA and other variables on CVD and all-cause mortality among men and women were estimated with the proportional hazard models. We assessed the contributions of VAs to the male–female mortality gap using hazard models that do and do not include groups of the predictors. Logistic models were used to assess the associations between VA and other biomarkers.

**Results:**

VAs were about twice as prevalent among men as among women. In both sexes, they were significantly associated with CVD and all-cause mortality independently of conventional risk factors. The highest hazard ratios (HRs) for CVD death were found for the runs of ventricular premature complexes (VPCs) HR = 2.45, 95% CI 1.63–3.68 for men and 2.75, 95% CI 1.18–6.40 for women. The mortality impacts of the polymorphic VPCs were significant among men only (HR = 1.50, 95% CI 1.08–2.07). VA indicators can potentially explain 12.3% and 9.1% of the male–female gaps in mortality from CVD and all causes, respectively. VAs were associated with ECG-registered ischemic problems and reported MI, particularly among men.

**Conclusions:**

VA indicators predicted mortality in older Muscovites independently of other risk factors, and have the potential to explain a non-trivial share of the excess male mortality. The latter may be related to more severe coronary problems in men compared to women.

## Introduction

In Russia, old-age mortality rates for CVD and all causes are very high, and the male–female mortality gap is among the largest in the world [1–3]. The life expectancy difference between women and men in Russia exceeds 10 years. Life expectancy decomposition shows that more than half of this difference is due to excess male mortality at ages 55+, half of which is, in turn, due to excess male mortality from CVD. While at young adult and midlife ages a substantial share of the Russian male mortality excess is attributable to alcohol and psychosocial stress, determinants of the male death toll at older ages, when heavy drinking is rare, remain unknown [4–7].

Earlier studies have found that conventional cardio-metabolic factors, such as hypertension, blood lipids, and obesity, can explain only a small share of the mortality excess in Russia, and of the male–female mortality gap [8–11]. Moreover, for other biomarkers, including specific characteristics of glucose metabolism, fibrinolysis, inflammation, and pulmonary function, only marginal differences have been found between Russia and other countries, or between Russian men and women [12–15].

Ventricular arrhythmia (VA) is an important contributor to the death toll in community-based populations [16, 17]. Frequent ventricular premature complexes are related to life-threatening arrhythmias [18]. It is generally assumed that VPCs threaten individuals with established CVD [19–21]. Several prospective studies have found elevated risks of MI, sudden cardiac death, and risks of death from coronary heart disease in apparently healthy subjects with frequent VPCs on standard ECG [22–24].

The growing use of long-term ECG (Holter) monitoring under normal daily life conditions in population-based studies allows for a more detailed and objective analysis of the links between VPCs and health outcomes. Using 1-h ambulatory ECG monitoring, the Framingham study found a more than two-fold increase in all-cause and CVD deaths among individuals with frequent VPCs [23]. Frishman et al. reported elevated risks of death and MI among a group of elderly individuals with ventricular tachycardia registered by 24-h ambulatory ECG recordings [25]. Sajadieh et al. analyzed 48-h Holter recordings of Copenhagen residents and found a two-fold risk of death or MI among subjects with frequent VPSs [26]. Another study that used data from the Cardiovascular Health Study (CHS, USA) detected increased risks of heart failure and mortality in elderly people from the general population with frequent VPCs [27].

Although these international studies have demonstrated the value of taking frequent VPCs into account when assessing the risk of CVD events or of dying, it is not yet clear whether frequent VPCs or other VA patterns can be considered independent predictors of death that add substantially to the conventional risk factors [28]. We are also not aware of any previous study that has examined the sex differences between VA patterns and their mortality impacts.

Our earlier analyses of data from the Stress, Aging, and Health in Russia (SAHR) study have found that elevated mortality risks and poor health are significantly associated with variables reflecting heart rate and heart rate variability [29, 30]. Our more recent study based on the same data showed that compared to women, men have a higher prevalence of major ECG abnormalities indicating ischemia and left ventricular hypertrophy [31]. These findings suggest that the male–female mortality gap could be linked to mechanisms of cardiac regulation and functioning.

This study uses the population-based SAHR data, including 24-h ambulatory ECG monitoring, multiple risk factors and markers, as well as seven-year mortality follow-up to examine whether VA contributes to the high CVD mortality and the large mortality gap between men and women in Russia.

## Materials and methods

### Data

This study is a secondary analysis of existing data from SAHR, a prospective population-based study of Moscow residents aged 55 and older. The study’s design, questionnaire, biomedical measures, and procedures for sampling, recruitment, and interviewing have been described in detail elsewhere [13, 29]. SAHR was approved by the Ethical Committee of the National Research Center for Preventive Medicine (Certificate #28/1, 22.11.2002) and by the Institutional Review Board at Duke University. All research was performed following the relevant guidelines and regulations. Before their inclusion in the sample, all SAHR participants provided written informed consent for participation and further analysis of the data.

The baseline SAHR survey was fielded between December 1, 2006, and June 30, 2009. The fieldwork and data processing were conducted jointly by the National Research Center for Preventive Medicine (NRCPM) in Moscow, Russia; the Max Planck Institute for Demographic Research in Rostock, Germany; and Duke University in Durham, USA.

The baseline SAHR sample includes 1800 individuals (961 women and 839 men) with an average age at baseline of 68.3 years (SD = 7.8, min = 55, max = 92). The ratio of the number of individuals who were interviewed and medically tested to the number of those who had been personally invited for participation in the study team (the response rate) was equal to 66%. In most cases, the protocol was administered at a hospital. The participants who were unable or unwilling to come to the hospital (8%) were interviewed and examined at home using the same protocol.

The baseline SAHR survey produced a wide range of reported and biomedical data, including information on anthropometry, handgrip strength, clinical and home blood pressure, markers based on routine blood tests, blood biochemistry based on venous blood samples, concentrations of stress hormones based on urine samples, Minnesota coded parameters of the 10-min 12-lead ECG, and characteristics of heart functioning from the long-term ambulatory (Holter) ECG.

### Ventricular arrhythmia and other ECG measures

24-h ECG monitoring was performed using the Schiller Holter system with 3-channel MICROVIT MT-101/200 digital devices [13]. All recordings were examined by trained cardiologists in semi-manual mode and were checked again by another cardiologist. Holter recordings with less than 17 h of valid ECG were excluded from the analysis. Analyzable data series were ultimately available for 1,732 individuals (96% of the sample). The mean ECG recording time was 22.7 h (SD = 1.1 h, min = 17 h, max = 24 h).

The following VA patterns were evaluated: isolated ventricular premature complexes and VPC runs. A ventricular premature complex was defined as a premature wide ventricular complex (QRS duration ≥ 120 ms) with abnormal morphology and without preceding atrial activity, followed by a compensatory pause. For each recording, the frequency of VPCs was computed by dividing their total count by the total duration of the Holter recording. Following previous studies, frequent VPCs were defined by dichotomous variables corresponding to the three lower limits of the frequency: ≥ 10/h, ≥ 30/h, and ≥ 100/h [25, 26, 32–34]. In our regression models, we used the VPCs frequency variable corresponding to the ≥ 10/h limit. This cut-off value was chosen after a preliminary analysis of the predictive power of proportional hazard regressions connecting CVD deaths with VPCs frequency indicator based on either 10/h, 30/h, or 100/h cut-offs. It appeared that the 10/h cut-off corresponded to the highest values of the post-estimation Harrell’s C both in men and women.

The complexity of the VPC patterns was expressed by the presence of VPC runs (≥ 3 subsequent VPCs). The VPC morphology was described by another dichotomous variable that expressed the multiformity (polymorphism) of the VPCs. The latter was defined as the existence of a difference in the axes of the wide QRS complexes of the VPCs in the same ECG channel.

Ischemic episodes on the Holter recordings were indicated by the ST-segment depression (≤ − 2 mm) lasting longer than one minute. The ST depression for individuals with permanent pacing (n = 9) and/or atrial fibrillation (n = 74) was not evaluated and was considered as the missing value.

We also used an indicator of probable coronary heart disease corresponding to  pathological Q-waves (major QQS) corresponding to Minnesota codes 1–1–1 to 1–2–7 in the 12-lead ECG.

### Other explanatory variables

Education is an important socio-demographic predictor of mortality. In the descriptive analysis, three categories of education were used: lower than secondary school, secondary school or the equivalent, and higher than secondary school (university or other tertiary-level institution). As individuals with lower education are relatively rare in the general Moscow population and the SAHR sample, our regression models used a dichotomous variable with higher education opposing a combined category of secondary and lower education.

The other explanatory variables were classic CVD risk factors (smoking, BMI, blood pressure, total cholesterol, triglycerides); markers of inflammation (high-sensitivity C-reactive protein (CRP), and interleukin-6 (IL-6)); and a self-reported (as “diagnosed by a doctor”) adverse CVD events, such as MI and stroke.

The data from the SAHR questionnaire allowed us to classify individuals as current, former, or never smokers. As the CVD and the all-cause death hazard patterns did not significantly differ between the latter two categories (preliminary analysis not shown here), our regression models included a dichotomous variable with categories of current smokers vs. never and former smokers.

Additional file 1: Table S1 defines the high-risk categories for the biomedical variables. Following our previous studies on SAHR data, we used established cut-points whenever possible. Due to the high (over 70%) prevalence of mild hypertension and overweight in the SAHR sample, we chose the higher of the available established cut-points that determine grade II hypertension (BP ≥ 160/100 mmHg) and obesity (BMI ≥ 30 kg/m^2^).

Following earlier SAHR-based papers, we used the sex-specific upper quintiles of the IL-6 distribution with the same values as those used in our previous studies [29–31].

Finally, we identified the major CVD events by reviewing the respondents’ reports of MI and stroke. Those reports were based on responses to the two questions that asked participants whether they had ever been told by a doctor that they had experienced MI or a stroke.

### Mortality follow-up

This study linked the baseline values of the VA variables with and without adjustment for certain sets of covariates with the following mortality. The mean length of the follow-up was equal to 7.4 years (SD = 2.2 years, min = 10 days, max = 9.6 years). The total exposure time was 12833 years (5555 for males and 7278 years for females). During this time, 416 deaths (271 men and 145 women) and 248 CVD deaths (166 men and 82 women) occurred.

### Analytic strategy

A descriptive table shows the major socio-demographic and biomedical characteristics for men and women and indicates whether the sex differences are statistically significant. Another table shows the prevalence of high-risk values of VA indicators on the Holter recordings, and also tests for the significance of the male–female differences. The two tabulations were done with post-stratification weights to adjust for differences in education (within each sex) between the SAHR sample and the general population of Moscow (according to the census of 2002). The significance of the male–female differences was checked with a Chi-square test.

We applied two proportional hazard models to assess the impacts of high-risk values of VA variables on mortality from CVD and all causes. In Model 1 (minimally-adjusted model), each VA predictor was used with adjustment for age and education. Model 2 (fully-adjusted model) additionally included the classic cardio-metabolic factors, as well as the reported history of MI and stroke. The fully-adjusted analysis allowed us to see whether the VA variables can predict the risk of death independently of the conventional cardio-metabolic factors. In all of the models, the mortality impacts of the predictors were quantified by hazard ratios (HRs).

To reveal any potential sex differences in associations between VA indices and mortality, the proportional hazard models were estimated separately for men and women. To check whether the strength of these associations differed by sex, we carried out an additional analysis of mortality from CVD and all causes with interactions between sex and the VA variables (not shown in the paper).

In the CVD mortality models, individuals who died from causes other than CVD were treated as censored as of the date of death. After estimating each model, we visually checked that the double logged survival probability curves on the logged time corresponding to different values of the covariates were reasonably parallel. We then checked that the fitted survival curves were well approximated by the Cox model curves. Finally, we confirmed that the Schoenfeld residual test returned no evidence of a violation of the proportional hazard assumption for specific variables, or globally.

After looking at the male–female differences in VA prevalence and assessing the links between VA and the death hazard for men and women, we assessed the VA indicators as mediators of the male–female gap in mortality from CVD and all causes. To do so, we applied a HR attenuation approach following our earlier SAHR-based study [30]. We assessed whether VA variables and other groups of factors accounted for the relationship between sex and mortality by comparing the HR values between hazard models that do and do not include each group of the predictors. This enabled us to compare the excess risk of being a male across five models: the minimally-adjusted model (1) including the male sex together with age, education, and sex; (2) age, education, and smoking; (3) age, education, and classic CVD risk factors; (4) age, education, reported MI and stroke, and ST depression; and (5) age, education, and VA variables. An additional model (6) includes all variables from models (1) to (5), and shows the ability of all predictors taken together to explain the male mortality excess.

We also carried out an additional analysis that linked CVD and all-cause mortality with biomedical variables other than VA. The results are presented in Additional file 1: Table S2.

To check whether there was a link in our data between VA and coronary problems, we connected the VA variables using logistic models to reported MI, major QQS, and ST depression (Additional file 1: Table S3).

All statistical analyses were carried out using Stata 15.0 (Stata Corp 2017).

## Results

### Description of the baseline sample

Table 1 shows that among Muscovites aged 55+, obesity and hypercholesterolemia are more prevalent in women, and that grade II hypertension is more prevalent in men. It also indicates that men are more likely than women to experience MI and stroke and that there are no significant differences by sex in the inflammation markers. The proportion of current or former smokers is shown to be much higher among men than among women. These findings are consistent with those of earlier studies [35, 36]. As expected for the Moscow population [13], levels of education are found to be high.

**Table 1:** Descriptive statistics for the baseline SAHR sample

Variable	Mean or prevalence		
	Males	Females	P for male-female difference
	n = 804	n = 928	
*Socio-demographic*			
Age	67.4 (8.4)^a^	69.5 (8.8)	< 0.001
Education (%)			
High	40.2	30.3	< 0.001
Middle	43.8	46.7	0.222
Low	16.0	22.9	< 0.001
*Classic CVD factors*			
Smoking (%)			
Never	32.6	82.4	< 0.001
Former	40.6	10.4	< 0.001
Current	26.8	7.1	< 0.001
Obesity (BMI ≥ 30.0 kg/m^2^) (%)	28.6	46.6	< 0.001
Grade II hypertension (BP ≥ 160/100 mmHg)	30.5	24.3	< 0.01
Total cholesterol ≥ 6.2 mmol/L	28.7	51.1	< 0.001
Triglycerides (TG ≥ 2.3 mmol/L)	9.1	7.7	0.313
*Inflammation*			
Interleukin-6 (IL-6 ≥ 2.0 pg/L for females, ≥ 2.4 pg/L for males) (%)	20.2	19.8	0.821
C-reactive protein (CRP ≥ 3.0 mg/L) (%)	31.3	35.0	0.099
*Reported CVD events*			
Myocardial infarction (%)	15.1	5.8	< 0.001
Stroke (%)	9.5	5.9	< 0.01

Adjusted for the difference in the educational composition of the general Moscow population

^a^Standard deviation is given in parentheses

Table 2 shows that 87.8% of men and 72.9% of women in the sample have at least one VPC on their Holter recordings. Elevated VPCs frequencies (≥ 10/h, ≥ 30/h, and ≥ 100/h) are found to be almost twice as prevalent among men as among women. Polymorphic VPCs are observed in 46.6% of men and 26.9% of women. VPC runs are found in 12.7% of men and 3.5% of women.

**Table 2:** Prevalence of ventricular arrhythmia on ECG recordings (in percent)

	Prevalence			
	Both sexes	Males	Females	P for male–female difference
		n = 804	n = 928	
*Ventricular arrhythmia*				
Any VPC	79.8	87.8	72.9	< 0.001
Freq VPC ≥ 10/h	17.3	23.0	12.4	< 0.001
Freq VPC ≥ 30/h	9.4	12.4	6.7	< 0.001
Freq VPC ≥ 100/h	3.5	4.9	2.3	< 0.01
Polymorphic VPC	36.0	46.6	26.9	< 0.001
VPC runs	6.2	9.5	3.5	< 0.001

Adjusted for the difference in the educational composition of the general Moscow population

### Links between mortality and ventricular arrhythmia

Table 3 shows the associations of the four VA variables with mortality from CVD (section A) and with mortality from all causes (section B). Besides, Additional file 1: Table S2 provides mortality HRs (all causes and CVD) for the classic CVD factors, inflammation markers, and reported MI and stroke derived from age-adjusted survival models with one factor/marker included in each model.

**Table 3:** The relationships between mortality from CVD (section A) and all causes (section B) and markers of ventricular arrhythmia. Cox regression hazard ratios

	Model 1			Model 2		
	HR	95% CI	*P*	HR	95% CI	*P*
*(A) CVD*						
Men						
VPC ≥ 10/h	1.537*	(1.110;2.129)	< 0.05	1.402*	(1.006;1.954)	< 0.05
Polymorphic VPC	1.477*	(1.079;2.023)	< 0.05	1.496*	(1.082;2.066)	< 0.05
VPC runs	2.614***	(1.763;3.876)	< 0.001	2.447***	(1.626;3.683)	< 0.001
Women						
VPC ≥ 10/h	2.076**	(1.251;3.445)	< 0.01	2.389**	(1.416;4.031)	0.001
Polymorphic VPC	1.281	(0.810;2.028)	0.290	1.171	(0.733;1.872)	0.509
VPC runs	2.213	(0.963;5.085)	< 0.1	2.746*	(1.178;6.403)	< 0.05
*(B) All causes*						
Men						
VPC ≥ 10/h	1.272	(0.976;1.658)	< 0.1	1.197	(0.915;1.566)	0.189
Polymorphic VPC	1.449**	(1.133;1.853)	< 0.01	1.481**	(1.153;1.902)	< 0.01
VPC runs	2.177***	(1.563;3.032)	< 0.001	2.119***	(1.508;2.977)	< 0.001
Women						
VPC ≥ 10/h	1.514	(0.995;2.304)	< 0.1	1.594*	(1.040;2.442)	< 0.05
Polymorphic VPC	1.228	(0.866;1.743)	0.250	1.107	(0.771;1.589)	0.582
VPC runs	1.902	(0.968;3.737)	< 0.1	2.187*	(1.105;4.331)	< 0.05

^*^p < 0.05; **p < 0.01; ***p < 0.001

Model 1: Adjusted for age and education

Model 2: Adjusted for age, education, smoking, grade II hypertension, obesity, total cholesterol, triglycerides, reported MI, and stroke

Table 3 demonstrates that there are significant links between all VA variables and elevated risks of death from CVD and all causes. The results of the minimally adjusted Model 1 are quite similar to the results of Model 2. Among men and women, the highest HR values for CVD are observed for VPC runs 2.4 (95% CI 1.6, 3.7) for men and 2.8 (95% CI 1.5, 5.1) for women in Model 2. The CVD mortality impacts of VPCs frequency ≥ 10/h are 2.39 (95% CI 1.42, 4.03) and 1.40 (95% CI 1.01, 1.95) in Model 2 for men and women, respectively. Finally, the mortality impacts of the polymorphic VPCs are found to be statistically significant in men only: 1.5 (95% CI 1.1, 2.0) in Models 1 and 2, respectively.

The all-cause mortality outcomes (Table 3, section B) generally mirror those for CVD. However, the point estimates of the HR values and the levels of statistical significance are slightly lower than those for CVD.

Results of regression models with the interaction between sex and VA variables (analysis not shown here) provide no evidence of a male–female difference in the mortality impacts of VA variables. A somewhat larger difference between the sexes (a greater impact in women) is seen for the VPCs frequency ≥ 10/h. but it does not reach statistical significance (*p* = 0.09).

### The contribution of ventricular arrhythmia to the male–female mortality gap

Table 4 presents a set of proportional hazard models for CVD mortality (section A) and all-cause mortality (section B) for men and women combined. In Model 1, the death hazard is linked to sex with adjustment for age and education only. This model shows the male-to-female HR (excess male mortality) in the absence of mediation. Models 2–5 allow us to see to what extent each of the groups of variables attenuates the maximal male-to-female HR of Model 1. Finally, Model 6 shows us to what extent all groups of variables together attenuate the maximal male-to-female hazard ratio of Model 1.

**Table 4:** Attenuation of the male-to-female hazard ratio by different groups of variables in proportional hazard models

*Sex*	Model 1	Model 2	Model 3	Model 4	Model 5	Model 6
	2.255*** (1.725–2.947)	1.923*** (1.457–2.538)	2.194*** (1.649–2.919)	2.054*** (1.564–2.698)	1.977*** (1.502–2.603)	1.549*** (1.141–2.105)
*(A) CVD*						
Smoking		2.431*** (1.762–3.355)				2.405*** (1.727–3.349)
Obesity			1.041 (0.785–1.361)			1.172 (0.878–1.565)
Grade II hypertension			1.264 (0.959–1.667)			1.237 (0.932–1.642)
Cholesterol			0.931 (0.704–1.230)			0.934 (0.705 – 1.236)
TG			1.108 (0.671–1.828)			1.192 (0.721 – 1.969)
Reported MI				1.470* (1.062–2.034)		1.375 (0.988–1.912)
Reported stroke				1.747** (1.236–2.470)		1.823** (1.287–2.581)
ST depression				1.506* (1.071–2.117)		1.473* (1.040–2.086)
VPC ≥ 10/h					1.381* (1.034–1.844)	1.333 (0.992–1.791)
Polymorphic VPC					1.256 (0.957–1.650)	1.179 (0.894–1.554)
VPC runs					2.196*** (1.518–3.180)	2.228*** (1.523–3.259)
N	1732	1732	1726	1727	1729	1720
Reduction of M/F HR compared to Model 1, %	-	14.7	2.7	8.9	12.3	31.3

*Sex*	Model 1	Model 2	Model 3	Model 4	Model 5	Model 6
	2.193*** (1.788–2.689)	1.932*** (1.564–2.386)	2.185*** (1.759–2.715)	2.084*** (1.693–2.565)	1.994*** (1.618–2.458)	1.704*** (1.352–2.148)
(B) All causes						
Smoking		2.058**** (1.600*–*2.647)				2.059**** (1.593*–*2.662)
Obesity			1.001 (0.805*–*1.245)			1.071 (0.857*–*1.339)
Grade II hypertension			1.136 (0.913*–*1.412)			1.108 (0.887*–*1.385)
Total cholesterol			1.000 (0.809*–*1.236)			0.986 (0.796 – 1.221)
Triglycerides			0.897 (0.595*–*1.354)			0.944 (0.625 – 1.426)
Reported MI				1.208 (0.920*–*1.586)		1.132 (0.859*–*1.492)
Reported stroke				1.549** (1.162*–*2.065)		1.565** (1.172*–*2.092)
ST depression				1.461** (1.110*–*1.922)		1.427* (1.079*–*1.885)
VPC ≥ 10/h					1.131 (0.894–1.431)	1.100 (0.866–1.397)
Polymorphic VPC					1.283* (1.040–1.582)	1.225 (0.991–1.515)
VPC runs					1.904*** (1.400–2.592)	1.947*** (1.422–2.666)
N	1732	1732	1726	1727	1729	1720
Reduction of M/F HR compared to Model 1, %	-	11.9	0.4	5.0	9.1	22.2

^*^p < 0.05; **p < 0.01; ***p < 0.001

Model 1: Adjusted for age and education

Model 2: Adjusted for age, education, and smoking

Model 3: Adjusted for age, education, obesity, grade II hypertension, total cholesterol, and triglycerides

Model 4: Adjusted for age, education, reported MI and stroke, and ST depression

Model 5: Adjusted for age, education, VPC ≥ 10/h, polymorphic VPC, and VPC runs

Model 6: Adjusted for age, education, smoking, obesity, grade II hypertension, total cholesterol, triglycerides, reported MI and stroke, ST depression, VPCs ≥ 10/h, polymorphic VPC, and VPC runs

After adjustment for age and education, the male-to-female HR is 2.3 (95% CI 1.7, 2.9) for CVD and 2.2 (95% CI 1.8, 2.7) for all-cause mortality. The largest shares of these gaps are attributable to smoking: 14.7% for CVD and 11.9% for all causes. The VA variables explain the second-largest shares: 12.3% for CVD and 9.1% for all causes. Finally, 8.9% and 5% of the male–female hazard ratio are attributable to the combined action of the history of MI, stroke, and ST depression for CVD and all causes of death, respectively. The contributions of the classic CVD risk factors are low.

Model 6 shows that all variables together reduce the initial male–female gap in the death hazard by 31.3% for CVD and by 22.2% for all causes, respectively.

### Associations between ventricular arrhythmia and coronary problems

The estimated logistic regression odds ratios (Additional file 1: Table S3) show the associations of the VA variables with coronary problems and ischemia. All of the VA indicators except polymorphic VPCs are statistically significantly associated with reported MI in men. Among men, all of the VA indicators are also strongly and statistically significantly associated with the major Q-wave abnormalities.

In our sample, the links between VAs and coronary problems are generally weaker in women than those in men. Although many point estimates for women in Additional file 1: Table S3 are elevated, respective associations do not reach statistical significance with the only exception for the association between VPCs frequency ≥ 10/h and ST depression.

## Discussion

We examined the prevalence, the mortality impacts, and the correlates of ventricular arrhythmia in a population-based sample of Moscow residents aged 55 or older, with an emphasis on the differences between men and women in a context characterized by high mortality and a huge mortality gap between men and women.

The present study has important advantages. The quality of VA diagnostics strongly depends on the duration of the ECG recordings. The use of 24-h Holter monitoring enabled us to identify a larger number of subjects with ventricular arrhythmia and a variety VA patterns than would have been possible using standard ECG, and to assess the true burden of VA [27, 37, 38]. For example, arrhythmia cannot be registered by a short-term ECG if it occurs only at night or during emotional or physical activity. That is why the Holter monitoring has higher sensitivity and reproducibility in VA diagnostics.

The availability of a broad range of biomarkers in SAHR allowed us to evaluate the prognostic importance of VA in the presence of many other risk factors. Despite its massive biomedical component, SAHR has a relatively large sample size. The seven-year mortality follow-up is sufficient to implement survival models and other analyses for each sex separately.

Our results on the prevalence of VPCs among Muscovites are consistent with the findings of earlier population-based studies. For both sexes, the prevalence of at least one VPC in SAHR was about 80%. In CHS, the VPC prevalence among subjects with a mean age of 71.9 was estimated at 82% [39]. Another study conducted in the 1990s reported a VPC prevalence of 80% among subjects aged 60–85 [34]. The Copenhagen Holter Study, which looked at 48-h Holter monitoring for individuals aged 55–75, reported a 95% prevalence [26]. These findings suggest very high prevalence of VPCs among older people in different countries.

Compared to the results of the Copenhagen Holter Study, our findings indicated that VPCs frequencies ≥ 30/h were more prevalent in men (12.4% vs. 8.8%) and slightly less prevalent in women (6.8% vs. 7.4%). In CHS, VPCs frequencies ≥ 15/h were found in 24.9% of men and 13.7% of women. These values are quite close to our results, with VPCs frequency ≥ 10/h found in 23% of men and 12.4% of women.

In our study, the prevalence of polymorphic VPCs for both sexes combined was equal to 36%. A similar prevalence value (38%) was found in the Bronx Longitudinal Aging Study [25]. Here, we are reporting for the first time the sex-specific prevalence of polymorphic VPCs in a population-based sample. It appears that this prevalence is 1.7 times higher in males than in females (47% vs. 27%). The only earlier study that reported the sex-specific prevalence of polymorphic VPCs was conducted on a (non-population-based) sample of hospital patients [38]. This study also found that polymorphic VPCs were about twice as prevalent among men as among women [40].

In our study, the prevalence of VPC runs for both sexes was equal to 6.3%. This value is between the corresponding values of 4% found by Fleg and Kennedy [34] and 7% found by the CHS [38]. Our findings further indicated that VPC runs were three times more frequent in men than in women. The same male–female ratio was observed in the CHS, the only earlier Holter-using population-based study that assessed the prevalence of VPC runs by sex. Interestingly, in both studies (analysis not shown here) the prevalence of the less threatening supraventricular arrhythmia was found to be about equal between men and women [39].

All comparisons of the prevalence figures should be interpreted  with caution due to inter-study differences in equipment and design. Nevertheless, we have to admit that our results do not provide any evidence that the prevalence of VA in Muscovites differs substantially from that in other populations.

Our proportional hazard models showed significant associations between VA and mortality from CVD and all causes. The strongest associations with HR values above two were observed for VPC runs in men and women and for VPCs frequency ≥ 10/h in women. Mortality from CVD and all causes was also found to be associated with polymorphic VPCs in men. Associations of VPCs frequency ≥ 10/h with all-cause mortality were shown to be marginally significant among men. Associations of polymorphic VPCs with all-cause mortality were found to be significant in men only.

Our findings regarding VPCs frequency are generally consistent with previous studies that found relationships between elevated VPCs frequency and death [23, 27], and between elevated VPCs frequency and death or MI [26]. None of the prior population-based studies has estimated the mortality impacts of VA patterns such as polymorphic VPCs or VPC runs. Only one earlier study based on a large clinical (non-population-based) sample assessed the relationship between polymorphic VPCs and mortality [40]. As in our study, the authors found a significantly elevated risk of death in individuals with multiform VPCs, but they did not perform this analysis by sex. Thus, our study is probably the first to show that polymorphic VPCs have a greater impact on mortality among men than among women.

The relationships between VA and mortality were found to be nearly the same in the minimally-adjusted and the multivariable-adjusted analyses. This result suggests that VA variables bear an additional risk of CVD and all-cause death even when the classic CVD factors and reported CVD events are controlled for. This result should be considered with full awareness of the part ischemic heart disease plays in the development of VA at older ages [41–43]. Our analysis of associations between VA variables and reported MI, major QQS, and ST depression at baseline also suggests the importance of pre-existing coronary conditions as risk factors of VA. Our results further indicate that for men aged 55–64 to 80+, the ischemic pathways of VA may be more important compared to women of the same age.

The inclusion of different biomarker groups in the hazard models allowed us to examine to what extent the male–female mortality gap attenuated in response to each of these groups. Smoking was found to account for the largest part of the gender difference. This is not surprising given the very large gender gap in smoking among Russians, especially at older ages [35, 36]. The VA variables showed their potential ability to explain the second-largest share of the male–female mortality gap for CVD (12.3%) and all causes (9.1%). MI, stroke, ECG-based ischemia, and the classic CVD factors were found to account for smaller portions of the gender gap. This is the first evidence that VA may be a significant contributor to excess male mortality. As comparative population-based data from other countries do not exist, we cannot determine how this finding compares to those elsewhere.

Noteworthy, we found a large male excess in the prevalence of VA indicators along with inconsistent male–female differences in relative risks associated with these indicators. For a direct assessment of contributions of the sex differences in the prevalence and the sex differences in respective mortality risks, one has to apply the full-scale mediation analysis [44–46]. This method is a more advanced one compared to the attenuation analysis used in our study. However, our data had not enough statistical power due to the small death numbers in some combinations of exposures.

Our study has also some other limitations. In SAHR data, there is no information about morphological changes in the myocardium as well as information from medical records concerning pre-existing ischemia, other medical diagnoses, and medications. Therefore, we were unable to evaluate in full the coronary status of subjects under study.

All of our biomarker measurements were carried out simultaneously. Therefore, our analysis of links between VA and coronary/ischemic markers cannot be considered causal.

## Conclusions

This study was probably the first to examine population-based differences between men and women in VA patterns and the corresponding mortality hazards. Our results show the predictive potential of VA indicators for death from CVD and all causes in a population. The mediation analysis showed that VA could contribute to the gaps in CVD and all-cause mortality between men and women. This is directly connected to the large gender gap in the prevalence of VA, which may, in turn, be related to the greater pre-existing coronary problems in Russian men compared to women.

We found no evidence for a difference in the prevalence of VA between the Russian sample and samples in earlier international studies. This confirmation of a “null hypothesis” is an unexpected but instructive finding given the exceptionally high level of CVD mortality in Russia.

## Supplementary Information

**Additional file 1. Table S1**: Cut-offs defining high-risk values for markers of biological risk. **Table S2**: Results of univariate age-adjusted proportional hazard models for socio-demographic characteristics, classic CVD factors, markers of inflammation, reported MI, and stroke. **Table S3**: Logistic regression odds ratios for the assessment of links between ventricular arrhythmia and reported MI, major Q-wave abnormalities, and ST depression.

## Data Availability

The data that support the findings of the study are available from the Stress Aging and Health in Russia study, but restrictions apply to the availability of these data. This is related to privacy protection and ethical restrictions imposed by the Institutional Review Board (IRB) for Clinical Investigations at the Duke University Health System (Durham, USA), the Independent Ethical Committee of the State Research Center for Preventive Medicine, Ministry of Health and Social Development of the Russian Federation (Moscow, Russia), and the Max Planck Society (Munich, Germany). The authors confirm that the data underlying the findings described in the manuscript are available to interested researchers upon submission of the Data Use Agreement and a short description of a scientific project to the responsible investigator: Dr. Vladimir M. Shkolnikov (shkolnikov@demogr.mpg.de).

## Abbreviations

CHS: Cardiovascular Health Study (USA)
CRP: C-reactive protein
CVD: Cardiovascular diseases
ECG: Electrocardiography/electrocardiogram
HR: Hazard ratio in a proportional hazard model
IL-6: Interleukin 6
Major QQS: Major Q-QS wave abnormalities
MI: Myocardial infarction
QRS: QRS complex on ECG
SAHR: Stress Aging and Health in Russia (a study)
SD: Standard deviation
ST depression: An electrocardiographic pattern with the ST segment being abnormally low
VA: Ventricular arrhythmia
VA: Ventricular arrhythmia
VPC: Ventricular premature complex on ECG
