# Supplementary material for: Prevalence, correlates, and mortality impacts of ventricular arrhythmia among older men and women: a population-based cohort study in Moscow
Source: BMC Cardiovasc Disord. 2021 Feb 8;21:80. doi: 10.1186/s12872-021-01883-0 (PMC7871639; doi:10.1186/s12872-021-01883-0)
Supplement: Supplementary file 1 — Additional file 1. Table S1: Cut-offs defining high-risk values for markers of biological risk. Table S2: Results of univariate age-adjusted proportional hazard models for socio-demographic characteristics, classic CVD factors, markers of inflammation, reported MI, and stroke. Table S3: Logistic regression odds ratios for the assessment of links between ventricular arrhythmia and reported MI, major Q-wave abnormalities, and ST depression. [file 12872_2021_1883_MOESM1_ESM.docx]

**Supplementary Table 1. Cut-offs defining high-risk values for markers of biological risk.**

|  | Mean | | High-Risk Cutoff | Reference |
| --- | --- | --- | --- | --- |
|  | Men | Women |  |  |
|  | (n=671) | (n=824) |  |  |
| **Conventional cardiovascular** |  |  |  |  |
| Systolic blood pressure (SBP, mmHg) | 145.3 | 142.4 | >160 | (1) |
| Diastolic blood pressure (DBP, mmHg) | 84.3 | 79.7 | >100 |  |
| Total Cholesterol (mmol/L) | 5.7 | 6.3 | >6.216 (240 mg/dL) | (2) |
| High-density liprotein (HDL, mmol/L) | 1.2 | 1.3 | <1.036 (40 mg/dL) |  |
| Triglycerides (mmol/L) | 1.3 | 1.4 | >2.26 (200 mg/dL) |  |
| Body mass index (BMI) | 27.6 | 29.8 | >30.0 | (3) |
| **Inflammation** |  |  |  |  |
| Interleukin-6 (IL-6,pg/mL) | 1.6 | 1.6 | >2.0 (M), >2.4 (F) | (4) |
| CRP (mg/dL) | 3.5 | 3.3 | >3 mg/L | (5) |

*Notes*

**(1)**  Gabb, G. M., Mangoni, A. A., Anderson, C. S., Cowley, D., et al. (2016) Guideline for the diagnosis and management of hypertension in adults—2016. Medical Journal of Australia, 205(2), 85-89. <https://doi.org/10.5694/mja16.00526>. **(2)** National Cholesterol Education Program (US). (2002) Expert Panel on Detection, Treatment of High Blood Cholesterol in Adults. Third report of the National Cholesterol Education Program (NCEP) expert panel on detection, evaluation, and treatment of high blood cholesterol in adults (adult treatment panel III) National Cholesterol Education Program, National Heart, Lung, and Blood Institute, National Institutes of Health. Circulation, 106(25), 3143–3421. **(3)** NHLBI (National Heart, Lung, and Blood Institute). (1998). Clinical guidelines on the identification, evaluation, and treatment of overweight and obesity in adults: executive summary. Bethesda, MD: National Institutes of Health. **(4)** Defined empirically by the highest quintile. **(5)** Krintus, M., Kozinski, M., Kubica, J., Sypniewska, G. (2014) Critical appraisal of inflammatory markers in cardiovascular risk stratification. Critical Reviews in Clinical Laboratory Sciences, 2014, 51(5), 263–279. <https://doi.org/10.3109/10408363.2014.913549>

**Supplementary Table 2. Results of univariate age-adjusted proportional hazard models for socio-demographic characteristics, classic CVD factors, markers of inflammation, reported MI and stroke.**

*A*) CVD

|  | Men | | Women | |
| --- | --- | --- | --- | --- |
|  | HR | 95% CI | HR | 95%CI |
| *Socio-demographic* |  |  |  |  |
| Middle and low education^#^ | 1.995*** | (1.415;2.814) | 1.450 | (0.026;2.271) |
| *Classic CVD factors* |  |  |  |  |
| Smoking^##^ | 2.813** | (1.996;3.964) | 1.133 | (0.342;3.757) |
| Obesity | 1.137 | (0.803;1.610) | 1.075 | (0.689;1.677) |
| Grade 2 hypertension | 1.238 | (0.894;1.713) | 1.812* | (1.137;2.888) |
| Total cholesterol | 1.056 | (0.748;1.489) | 0.781 | (0.502;1.214) |
| Triglycerides | 1.270 | (0.729;2.211) | 0.97 | (0.352;2.669) |
| *Inflammation* |  |  |  |  |
| Interleukin – 6 | 2.643** | (1.930;3.621) | 1.258 | (0.749;2.114) |
| CRP | 1.839** | (1.344;2.517) | 1.475 | (0.935;2.326) |
| *Major CVD events* |  |  |  |  |
| Reported MI | 1.657** | (1.161;2.365) | 1.934* | (1.012;3.696) |
| Reported stroke | 1.795** | (1.197;2.692) | 2.912** | (1.631;5.199) |
| *Ischemia* |  |  |  |  |
| ST depression | 1.581* | (1.051;2.378) | 1.813 | (0.90;3.320) |

*B*) All causes

|  | Men | | Women | |
| --- | --- | --- | --- | --- |
|  | HR | 95% CI | HR | 95%CI |
| *Socio-demographic* |  |  |  |  |
| Middle and low education^#^ | 1.732** | (1.332;2.250) | 1.653** | (1.177;2.320) |
| *Classic CVD factors* |  |  |  |  |
| Smoking^##^ | 2.321*** | (1.766;3.050) | 1.521 | (0.751;3.081) |
| Obesity | 1.029 | (0.780;1.358) | 1.081 | (0.775;1.508) |
| Grade 2 hypertension | 1.103 | (0.850;1.431) | 1.632** | (1.139;2.338) |
| Total cholesterol | 1.091 | (0.836;1.424) | 0.875 | (0.629;1.218) |
| Triglycerides | 1.041 | (0.657;1.649) | 0.726 | (0.319;1.651) |
| *Inflammation* |  |  |  |  |
| Interleukin – 6 | 2.185*** | (1.696;2.815) | 1.458 | (0.992;2.141) |
| CRP | 1.795*** | (1.404;2.296) | 1.563** | (1.115;2.192) |
| *Major CVD events* |  |  |  |  |
| Reported MI | 1.345 | (1.000;1.810) | 1.520 | (0.868;2.663) |
| Reported stroke | 1.693** | (1.218;2.354) | 1.858* | (1.102;3.134) |
| *Ischemia* |  |  |  |  |
| ST depression | 1.404* | (1.002;1967) | 1.931** | (1.215;3.068) |

*Notes*

^#^Reference group: higher education

^###^Reference group: non-smokers and former smokers

*p<0.05, **p<0.01, ***p<0.001

**Supplementary Table 3. Logistic regression odds ratios for the assessment of links between ventricular arrhythmia and reported MI, major Q-wave abnormalities, and ST depression.**

|  | Reported MI | Major QQS | ST depression |
| --- | --- | --- | --- |
|  |  | Men |  |
| VPC ≥10/hour | 1.66*(1.08;2.59) | 2.53**(1.42;4.51) | 1.11(0.66;1.87) |
| Polymorphic VPCs | 1.44(0.95;2.17) | 2.06*(1.15;3.69) | 1.28(0.81;2.03) |
| VPC runs | 1.91*(1.08;3.39) | 2.84**(1.42;5.72) | 1.40(0.86;2.29) |
|  |  | Women |  |
| VPC ≥10/hour | 1.77(0.85;3.71) | 1.63(0.70;3.81) | 2.08*(1.09;3.96) |
| Polymorphic VPC | 1.34(0.71;2.52) | 2.00(1.02;3.93) | 1.58(0.91;2.75) |
| VPC runs | 1.08(0.25;4.77) | 1.58(0.36;6.89) | 1.48(0.75;2.90) |

*Notes*

The major Q-wave abnormalities and ST depression are defined in the Materials and Methods (sub-section Ventricular arrhythmia and other ECG measures).

*p<0.05, **p<0.01, ***p<0.001
